# Insulin Requirement for Gestational Diabetes Control Is Related to Higher Vitamin D Levels up to 1 Year Postpartum: A Prospective Cohort Study

**DOI:** 10.3390/antiox11112230

**Published:** 2022-11-11

**Authors:** María Molina-Vega, María José Picón-César, Fuensanta Lima-Rubio, Carolina Gutiérrez-Repiso, Teresa María Linares-Pineda, María Suárez-Arana, Ana María Fernández-Ramos, Francisco J. Tinahones, Sonsoles Morcillo

**Affiliations:** 1 Departamento de Endocrinología y Nutrición, Hospital Universitario Virgen de la Victoria, 29010 Málaga, Spain; fjtinahones@uma.es (F.J.T.); sonsoles.morcillo@ibima.eu (S.M.); 2 Laboratorio de Investigación Biomédica de Málaga, Hospital Universitario Virgen de la Victoria, 29010 Málaga, Spain; santi.lima@hotmail.com (F.L.-R.); carogure@hotmail.com (C.G.-R.); teresamaria712@gmail.com (T.M.L.-P.); 3 Centro de Investigación Biomédica en Red (CIBER) de Fisiopatología de la Obesidad y Nutrición, Instituto Salud Carlos III, 28029 Madrid, Spain; 4 Departmento de Obstetricia y Ginecología, Hospital Regional Universitario de Málaga, IBIMA, 29009 Málaga, Spain; dramariasuarez@gmail.com; 5 Departamento de Análisis Clínicos, Hospital Universitario Virgen de la Victoria, 29010 Málaga, Spain; anam.fernandez.ramos.sspa@juntadeandalucia.es

**Keywords:** vitamin D, pregnancy, gestational diabetes mellitus, insulin

## Abstract

Vitamin D deficiency is highly prevalent in pregnant women and has been related to a higher risk of gestational diabetes mellitus (GDM). The aim of this study is to analyze vitamin D status evolution in a population of pregnant women with and without GDM. Two-hundred women were included from January 2019 to February 2022 as follows: Control group -CG-, Lifestyle group -LG- (GDM not requiring insulin), and Insulin group -IG- (GDM requiring insulin). Visits were carried out at baseline, antenatal, postpartum, and 1 year after birth. Vitamin D levels, weight, and insulin resistance were measured at every visit. Data about the season, vitamin D supplementation, Mediterranean diet adherence, and physical activity were included. In the three groups, 134 women were included in the CG, 43 in the LG, and 23 in the IG. Vitamin D levels were similar among the groups at baseline, but they were significantly higher in the LG and IG in comparison with the CG at the antenatal visit and significantly higher in the IG vs. CG and LG at the postpartum and 1 year after birth visits. Vitamin D levels were independently related to vitamin D supplementation and the season at baseline, to the season and belonging to the LG or IG at the antenatal visit, and were only independently associated with belonging to the IG at postpartum and 1 year after birth visits. In conclusion, in our population, women with GDM requiring insulin had higher levels of vitamin D in comparison with those not requiring insulin and healthy controls at postpartum and 1 year after pregnancy. Requiring insulin during pregnancy seems to be a factor that independently determines the levels of vitamin D until 1 year after birth. More studies are required to reproduce these data in other populations and to elucidate the mechanisms underlying these findings.

## 1. Introduction

Vitamin D is now recognized as a hormone and is mainly obtained by photosynthesizing in the skin but also from dietary sources. In order to produce the biologically active form, two consecutive enzymatic hydroxylation reactions are required, in the liver and kidney, resulting in 25-hydroxyvitamin D (25-OH-D; calcidiol) and 1α,25 dihydroxy vitamin D (1,25-OH2-D; calcitriol), respectively [1]. Classically, vitamin D has been associated with bone health and phosphor–calcium homeostasis. However, in the last decades, novel actions of vitamin D in the body have been suggested, including xenobiotic detoxification, oxidative stress reduction, neuroprotective functions, antimicrobial defense, immunoregulation, anti-inflammatory/anticancer actions, and cardiovascular benefits [1]. In addition, its relationships with multiple diseases, such as diabetes, colorectal cancer, and multiple sclerosis, among others, have been explored [2]. The important role of vitamin D in health makes the low levels of vitamin D in the population a matter of some concern, as the prevalence of vitamin D deficiency (25-OH-D < 20 ng/mL) in Europe ranges from 20 to 80%, and severe deficiency (25-OH-D < 12 ng/mL) reaches >10% [3].

Vitamin D has been linked to type 2 diabetes mellitus (T2DM), with those subjects with lower levels of vitamin D having a higher risk of developing T2DM according to data from studies in different cohorts [4,5]. Vitamin D has been suggested to favor β-cell function and to enhance insulin sensitivity by different mechanisms: the interaction with calcium flux-regulating receptors of β-cells, the reduction of the renin–angiotensin system hyperactivity, the stimulation of insulin receptor expression, the activation of the peroxisome proliferator-activated receptor delta (PPAR-δ), the deactivation of inflammatory cytokines associated with insulin resistance and the promotion of calbindin expression, which confers apoptosis protection [6]. Furthermore, as oxidative stress due to the imbalance between the reactive oxygen species (ROS) production and the cellular antioxidant system in an hyperglycemic environment results in the development of diabetes, the antioxidant effect of vitamin D could also influence the relationship between vitamin D and T2DM [7]. Vitamin D has been shown to ameliorate oxidative stress, through the upregulation of proteins such as the nuclear factor erythroid 2–related factor 2 (Nrf2) and the antioxidant enzymes glutathione peroxidase and reductase, among others [8]. Focusing on gestational diabetes mellitus (GDM), on one side, those women with GDM, especially when requiring insulin for treatment, have been found to have greater plasma levels of oxidative stress during the whole pregnancy in comparison to pregnant women without GDM [9]. On the other side, vitamin D supplementation in women with GDM has been reported to attenuate inflammation and oxidative stress [10].

It has been estimated that 54% of pregnant women have 25-OH-D < 20 ng/mL, and 20% have levels < 10 ng/mL [11]. Low levels of vitamin D during pregnancy have been related to higher risk of preeclampsia, cesarean section indication, preterm birth, low birth weight, and being small for gestational age [12]. Some studies have reported a higher risk of developing GDM among those women with lower levels of 25-OH-D [13,14], even independently of the body mass index (BMI) effect [15]. However, other authors failed to find any relationship between vitamin D status and GDM risk [16,17] or any benefit from achieving vitamin D sufficiency through supplementation for GDM prevention [18]. In a recent study, even high early pregnancy 25-OH-D levels were associated with a greater risk of GDM [19].

The aim of this study is to analyze the vitamin D status in those women with GDM in comparison to healthy controls and the possible factors influencing vitamin D levels during pregnancy, postpartum, and until 1 year after birth.

## 2. Materials and Methods

### 2.1. Study Population

From January 2019 to February 2022, 200 pregnant women who attended the Diabetes and Pregnancy Unit of Hospital Virgen de la Victoria de Málaga, after a positive 50 g oral glucose load, were included to participate in the project “Effect of gestational diabetes on the epigenome of the mother and the offspring during the first 1000 days. Identification of potential epigenetic biomarkers of risk of DM2 and obesity” (funded by Instituto de Salud Carlos III, Spain; PI18/01175). All participants provided written informed consent.

Women aged 18–45 years in the 2nd or 3rd trimester of pregnancy were included. The existence of GDM diagnosis at <14 weeks of pregnancy or multiple pregnancy were exclusion criteria.

### 2.2. Study Protocol

The diagnostic 100 g oral glucose tolerance test (OGTT) was performed on all women. GDM was diagnosed according to National Diabetes Data Group (NDDG) criteria. The thresholds for the 100 g OGTT were 105 mg/dL for fasting glucose and 190 mg/dL, 165 mg/dL, and 145 mg/dL at 60, 120, and 180 min, respectively. GDM was diagnosed when glucose met or exceeded these levels at 2 or more time points [20].

Those women with a negative 100 gr OGTT were classified as controls (“Control group”). In women diagnosed with GDM, lifestyle changes and self-blood glucose monitoring (SBGM) at fasting and 1 h postprandrial after breakfast, lunch, and dinner (Bayer, Contour^®^ Next Glucose test strips, XT or USB meters) were recommended. After 1 week, glycemic controls were analyzed by the endocrinologist. If ≥2 glucose fasting values were ≥95 mg/dL and/or 1 h postprandial ≥ 140 mg/dL despite lifestyle changes, the addition of insulin therapy was indicated. According to the requirement or not of insulin therapy, women were classified as “Lifestyle group” or “Insulin group”.

The study protocol includes a baseline visit (at the moment of performance of 100 gr OGTT, at 24–30 weeks of pregnancy), an antenatal visit (at 36–37 weeks of pregnancy), a postpartum visit (8–10 weeks after birth) and a 1-year visit (1 year after birth). Blood samples were collected at every visit, after a 12 h fast. At the baseline and 1-year visits, patients completed the Mediterranean Diet (MD) Adherence survey adapted from [21], the Global Physical Activity Questionnaire (GPAQ) adapted from [22] and a structured interview in order to obtain data about associated nutritional supplementation (including vitamin D supplementation) or kind of feeding, among other data. Using data from GPAQ included in the section “Travel to and from places”, which entails sunlight exposure, we calculated the variable minutes of walking/using a bicycle per day. Height was measured at the baseline visit, while weight was measured at every visit.

Antecubital venous blood samples were collected after a 12 h fast at 8 a.m. at every visit. Glucose levels were analyzed with Dimension Vista Analyzer (Siemens AG) using the glucose oxidase method. HbA1c was determined by HPLC (high-performance liquid chromatography) by the ADAMS A1c HA-8180V Analyzer (Menarini). Serum 25(OH)D levels were determined by Enzyme-Linked ImmunoAssay (ELISA) kit (Immundiagnostik, Bensheim, Germany). Vitamin D levels were classified as a deficiency when 25-OH-D < 20 ng/mL and insufficiency if 25-OH-D ranged 21–29 ng/mL, according to the Endocrine Society Guideline [23]. Insulin levels were measured by a radioimmunoassay method using BioSource International Inc. (Camarillo, CA, USA). We calculated the homeostasis model assessment of insulin resistance index (HOMA-IR) and β-cell function (HOMA-B) as described by Matthews et al. [24].

Regarding seasons, depending on the date of every visit, we considered winter from 21st of December to 20th of March, spring from 21st of March to 20th of June, summer from 21st of June to 20th of September, and autumn from 21st of September to 20th of December.

### 2.3. Statistical Analysis

Data are shown as mean ± standard deviation or percentages. Comparisons between the quantitative data were performed by ANOVA test while comparisons between the qualitative data were tested by chi-aquared test. Multivariate linear regression analyses were performed to determine the association between 25-OH-D in every visit and the clinical and analytical variables. Those variables previously described as influencing 25-OH-D levels or those associated with vitamin D levels were included in the multivariate analysis in univariate linear regression analysis. Dummy variables were performed to introduce group and season as independent variables in the linear regression models.

Results were considered significant if *p* < 0.05. Statistical analysis was performed with SPSS (15.0 version for Windows; SPSS, Chicago, IL, USA).

### 2.4. Ethics

This study was reviewed and approved by the Ethics and Research Committee of Virgen de la Victoria University Hospital, Málaga, Spain and was conducted according to the principles of the Declaration of Helsinki. The participants (who were all volunteers) provided signed consent after being fully informed of the study goal and its characteristics.

## 3. Results

### 3.1. Comparison between Groups from Baseline to 1 Year after Birth

**Figure 1:**
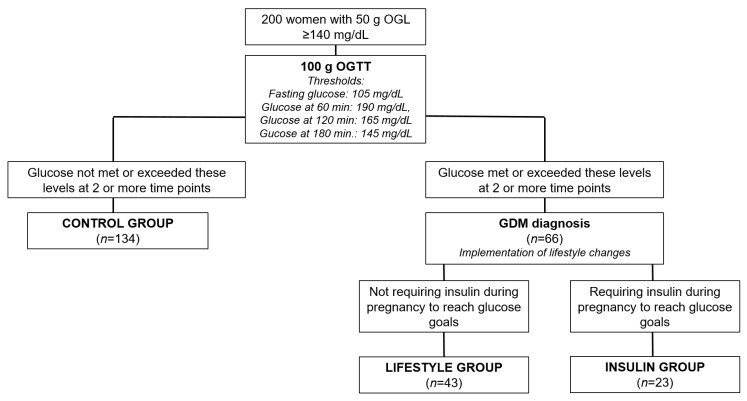
Flowchart. OGL: oral glucose load; OGTT: oral glucose tolerance test; GDM: gestational diabetes mellitus. GDM was diagnosed by the 100 gr OGTT, when glucose met or exceeded thresholds at 2 or more time points: 105 mg/dL for fasting glucose and 190 mg/dL, 165 mg/dL, and 145 mg/dL at 60, 120, and 180 min, respectively.

A total of 200 pregnant women were included in this study. The flowchart presenting the procedure for assigning patients to the study groups is shown in Figure 1.

The mean gestational age at the baseline visit was 27.5 ± 1.8 weeks, and the mean age was 33.2 ± 5.2. Mean 25-OH-D levels were 25.3 ± 9.2, with 28.3% of our population having deficiency and 44.4% insufficiency.

In Table 1, the characteristics of the study population and comparisons between groups at baseline, at the antenatal visit, at the postpartum visit and at 1 year after birth are shown. The 25-OH-D levels were similar between groups at baseline, but at antenatal, the levels in GDM women (independently of treatment) compared to controls were significantly higher and continued to be significantly higher in the Insulin group at postpartum and 1 year after birth in comparison to the Lifestyle group and Control group. Women in the Insulin group had a higher BMI in comparison with the other groups. This significant difference was maintained at postpartum but not 1 year after birth. However, women in the Control group were those who gained more weight during pregnancy. Similarly, women in the Insulin group had more insulin resistance (according to HOMA-IR), higher fasting insulin levels, and worse glucose metabolism parameters (higher fasting glucose and HbA1c) at baseline, antenatal, and postpartum visits, becoming similar to the other groups at 1 year after birth. β-cell function (according to HOMA-B) was similar between groups in all visits, except at the antenatal visit, when the Insulin group patients had a significantly higher HOMA-B. We did not find any differences regarding seasons.

No women were receiving vitamin D supplementation at postpartum or 1 year after birth.

### 3.2. Factors Related to Vitamin D Levels at Every Time Point

In Table 2, the multivariate linear regression analysis for 25-OH-D levels at every time point is shown. At baseline, 25-OH-D levels were independently and directly related to vitamin D supplementation and to the season (summer). At the antenatal visit, 25-OH-D levels were directly associated with belonging to the Lifestyle and Insulin group and to the season (summer and autumn). At postpartum and 1 year after birth, 25-OH-D was only directly related to having belonged to the Insulin group. In all multivariate analyses, age, weight (represented by BMI or weight gain), insulin resistance, and season were included. In addition, at baseline, MD adherence score and vitamin D supplementation were included as they were different between groups and significantly related to 25-OH-D levels in bivariate linear regression analysis (data not shown).

## 4. Discussion

In this prospective cohort study, including 200 pregnant women with or without GDM, it was found that, despite having similar 25-OH-D levels at baseline, those requiring insulin presented significantly higher levels of 25-OH-D at the antenatal visit, at postpartum, and even 1 year after birth. When analyzing possible factors related to 25-OH-D levels, we found that, although at baseline 25-OH-D was associated with vitamin D supplementation or season, at postpartum and 1 year after birth, only having received insulin during pregnancy was independently related to 25-OH-D levels.

Regarding the vitamin D status in our population, insufficiency prevalence was higher than previously reported by Karras et al. [25] for a Mediterranean population of pregnant women, while we found a vitamin D deficiency prevalence that was in the lower limit of the range previously described. They found a prevalence of 9.3 to 41.4% for insufficiency (in our population 44.4%) and 22.7 to 90.3% for deficiency (in our population 28.3%). Our prevalence of vitamin D insufficiency and deficiency was higher than that observed by Rodríguez et al. [26] in a Spanish population of pregnant women that was 31% and 18%, respectively, for insufficiency and deficiency.

Season (summer/spring), latitude, age, social class, race, skin color, dressing pattern, BMI, gestational age, smoking status, physical activity, and use of vitamin D supplements have been identified as independent determinants of 25-OH-D concentration in pregnant women [27,28]. In fact, vitamin D supplementation and season (summer) were the factors independently related to 25-OH-D levels in our population at baseline. Season (summer/autumn) also determined 25-OH-D levels at the antenatal visit, in addition to belonging to the Lifestyle group and to the Insulin group. However, the only factor independently related to 25-OH-D concentrations at the postpartum visit and 1 year after birth belonged to the Insulin group.

The relationship between vitamin D and diabetes has been widely studied regarding the effect of vitamin D on β-cell function and insulin sensitivity [6]. In addition, many authors have analyzed the influence of the status of vitamin D on T2DM and GDM incidence [4,5,13,14,15,16,17] or the effect of vitamin D supplementation in the prevention and treatment of T2DM and GDM [18,27,28,29]. In a recent review by Zhu et al. [30], 16 studies analyzing the association between vitamin D deficiency (10 at <20 weeks of pregnancy, 4 at >20 weeks of pregnancy, and 2 at both periods) and the risk of GDM were included. Eight studies found a higher risk of developing GDM when vitamin D was deficient at <20 weeks of pregnancy, while in four they failed to confirm this association. When vitamin D deficiency at >20 weeks of pregnancy was taken into account, four studies observed a higher risk of GDM in those women with vitamin D deficiency while two studies did not. Contrary to what was previously reported, Yong et al. [19] observed that a higher risk of GDM in those women with higher levels of vitamin D at early pregnancy had a greater risk of developing GDM. Although the results of this study are similar to those found by us, as they related higher vitamin D levels with GDM, our data differ from all other data that we have found in the literature. In fact, neither vitamin D levels nor the prevalence of vitamin D deficiency were different between women with or without GDM in our population. We observed differences after beginning the treatment and, to our knowledge, nobody has studied the possible effect of diabetes therapy on vitamin D. In our population, at the antenatal visit, those women with GDM had significantly higher levels of 25-OH-D than the controls. In addition, in both postpartum and 1 year after pregnancy, 25-OH-D levels were higher in GDM women requiring insulin in comparison with women who only required lifestyle changes to achieve adequate metabolic control of GDM and to non-GDM controls, although they were similar to the other groups in the rest of the parameters studied. The question is: how can insulin therapy during pregnancy influence the 25-OH-D levels even 1 year after the withdrawal of the treatment?

It is known that during pregnancy, an insulin-resistant state happens in order to increase glucose availability in the mother for transferring to the fetus [31,32]. In response to this, and to avoid excessive hyperglycemia and glucose intolerance, important adaptations occur in β-cell function, such as increased proliferation and glucose-stimulated insulin secretion [31,32]. However, in the presence of some predisposing factors such as maternal obesity or high weight gain during pregnancy, these adaptations cannot be adequately performed and GDM appears [31,32]. On the one hand, vitamin D could contribute to these adaptations as it has been found to improve β-cell function and favor insulin secretion [6]. On the other hand, some studies have demonstrated a significant improvement in insulin resistance and β-cell function in newly diagnosed T2DM patients treated with short-term intensive insulin therapy [33,34,35,36]. Taking all of the above into account, perhaps some changes in lifestyle and, in particular, insulin therapy, may help the function of β-cells in pregnant women not requiring the effect of vitamin D as much in comparison with women without GDM who have not been advised to take any action to improve physiologic insulin resistance in pregnancy and whose β-cells are working hard to balance it. Although this explanation might be meaningful during pregnancy, it does not seem to be applicable at postpartum or 1 year after birth.

The higher vitamin D levels in women with GDM in our population could be related to a higher motivation to adopt lifestyle recommendations, including more physical activity and, consequently, higher sunlight exposure, which contributes to vitamin D synthesis. It has been shown that pregnancy is a period in women’s’ lives when they are highly motivated to acquire a healthy lifestyle [37]. The concern related to the GDM diagnosis could increase this motivation. Ørtenblad et al. [38], reported that women diagnosed with GDM were highly motivated to take preventive initiatives and maintain them for up to 5 years after postpartum. However, a higher adherence to a Mediterranean diet or higher physical activity was not found in women with GDM in comparison with controls 1 year after birth.

Other factors that have not been considered in this study may be related to this increase in vitamin D levels. For example, some authors have shown how insulin can regulate gene expression by epigenetic mechanisms. On the one hand, Peng IC et al. recently showed how insulin treatment regulated the gene expression of genes related to lipogenesis and cell proliferation through DNA methylation [39]. On the other hand, other work performed in a human hepatocyte cell line (HepG2) demonstrated that insulin treatment resulted in a significant reduction in AQP9 expression mediated by epigenetic modifications at the promoter [40]. Could insulin therapy induce some epigenetic changes in genes related to vitamin D metabolism, influencing vitamin D levels in the long term? Further studies should be conducted to answer this issue.

As a strength of our work, we can highlight that this is the first study reporting these findings, the proper sample size, being prospective, and the follow-up until 1 year after birth. As a weakness, we can mention that we do not have enough data regarding diet and sunlight exposure at every time point and that, despite a representative sample size at the beginning, it decreased during the time of study.

## 5. Conclusions

In a population of pregnant women, those who required insulin therapy for the control of GDM presented higher levels of vitamin D than those women with GDM only requiring lifestyle changes and non-GDM women. Although 25-OH-D levels at baseline were observed to be related to some previously described factors such as vitamin D supplementation or season, at postpartum and 1 year after birth, only those belonging to the group requiring insulin were independently related to 25-OH-D concentrations. More studies are needed in order to reproduce these findings and to clarify the possible physiopathologic mechanisms explaining them.

## Figures and Tables

**Table 1:** Comparison between groups at every time point.

	Control	Lifestyle	Insulin	*p*
**Baseline (n)**	134	43	23	200
Age (years)	32.8 ± 5.3	34.5 ± 4.4	33.2 ± 5.2	0.214
BMI before pregnancy (kg/m^2^)	27.1 ± 6.5 ^a^	26.5 ± 5.8 ^a^	31.3 ± 6.0 ^b^	**0.008**
Fasting glycemia (mg/dL)	77.9 ± 11.3 ^a^	80.4 ± 9.9 ^a^	93.9 ± 9.2 ^b^	**<0.001**
Fasting insulin (µUI/mL)	9.7 ± 5.1 ^a^	11.8 ± 8.9 ^a^	17.2 ± 9.2 ^b^	**<0.001**
HbA1c (%)	5.1 ± 0.3 ^a^	5.1 ± 0.4 ^a^	5.5 ± 0.5 ^b^	**<0.001**
25-OH-D (ng/mL)	24.6 ± 8.5	27.6 ± 11.9	25.3 ± 7.3	0.224
25-OH-D categories (%) -Deficiency (<20 ng/mL) -Insufficiency (20–29.9 ng/mL) -Sufficiency (≥30 ng/mL)	28.4 49.1 22.5	32.5 25.0 42.5	21.7 52.2 26.1	0.053
Vitamin D supplements (%) -No -Yes	82.8 17.2	76.7 23.3	69.6 30.4	0.283
Vitamin D daily dose (IU)	608.7 ± 472.8	520.0 ± 168.6	428.5 ± 179.9	0.525
HOMA-IR	2.1 ± 1.2 ^a^	2.7 ± 2.3 ^b^	4.4 ± 2.5 ^c^	**<0.001**
HOMA-B	192.9 ± 162.7	152.1 ± 81.1	165.5 ± 62.2	0.412
MD adherence score	7.7 ± 1.9 ^a^	7.6 ± 1.9 ^a,b^	6.5 ± 2.2 ^b^	**0.030**
Daily walking/cycling (min.)	51.8 ± 72.7	37.9 ± 43.4	42.9 ± 57.1	0.453
Smoking during pregnancy (%) -No -Yes	92.5 7.5	90.5 9.5	78.3 21.7	0.099
Season (%) -Winter -Spring -Summer -Autumn	38.1 23.9 14.2 23.9	32.6 23.3 25.6 18.6	34.8 34.8 17.4 13.0	0.535
**Antenatal visit (n)**	66	31	18	115
Weight gain in pregnancy (kg)	12.0 ± 5.0 ^a^	6.9 ±5.5 ^b^	9.1 ± 5.8 ^b^	**<0.001**
Fasting glycemia (mg/dL)	76.8 ± 13.9	74.2 ± 11.8	80.0 ± 11.1	0.318
Fasting insulin (µUI/mL)	14.6 ± 19.7 ^a^	10.6 ± 6.9 ^a^	43.0 ± 25.1 ^b^	**<0.001**
HbA1c (%)	5.4 ± 0.3 ^a^	5.4 ± 0.3 ^a^	5.6 ± 0.3 ^b^	**0.010**
25-OH-D (ng/mL)	24.4 ± 9.5 ^a^	29.3 ± 13.4 ^b^	32.1 ± 9.8 ^b^	**0.013**
25-OH-D categories (%) -Deficiency (<20 ng/mL) -Insufficiency (20–29.9 ng/mL) -Sufficiency (≥30 ng/mL)	34.9 36.5 28.6	25.9 18.5 55.6	6.3 12.5 81.3	**0.002**
HOMA-IR	3.1 ± 5.9 ^a^	1.9 ± 1.3 ^a^	8.6 ± 5.2 ^b^	**<0.001**
HOMA-B	310.8 ± 755.2 ^a^	258.5 ± 718.67 ^a^	1092.7 ± 844.3 ^b^	**0.001**
Season (%) -Winter -Spring -Summer -Autumn	21.2 31.8 24.2 22.7	25.8 32.3 19.4 22.6	33.3 11.1 50.0 5.6	0.133
**Postpartum visit (n)**	51	26	16	93
BMI (kg/m^2^)	27.8 ± 5.9 ^a^	26.2 ± 5.0 ^a^	31.8 ± 5.1 ^b^	**0.009**
Fasting glycemia (mg/dL)	81.7 ± 6.7 ^a^	85.4 ± 14.2 ^a,b^	90.1 ± 6.5 ^b^	**0.007**
Fasting insulin (µUI/mL)	7.2 ± 5.3 ^a^	5.4 ± 3.3 ^a^	12.1 ± 9.9 ^b^	**0.002**
HbA1c (%)	5.2 ± 0.3 ^a^	5.2 ± 0.4 ^a^	5.4 ± 0.3 ^b^	**0.041**
25-OH-D (ng/mL)	25.2 ± 6.8 ^a^	26.8 ± 5.8 ^a^	29.8 ± 4.6 ^b^	**0.041**
25-OH-D categories (%) -Deficiency (<20 ng/mL) -Insufficiency (20–29.9 ng/mL) -Sufficiency (≥30 ng/mL)	22 54 24	11.5 61.5 26.9	0 50 50	0.120
HOMA-IR	1.5 ± 1.1 ^a^	1.1 ± 0.8 ^a^	2.7 ± 2.3 ^b^	**0.001**
HOMA-B	147.7 ± 100.2	106.2 ± 68.5	163.7 ± 121.9	0.115
Kind of feeding (%) -Breastfeeding -Artificial feeding -Mixed feeding	64.7 21.6 13.7	61.5 15.4 23.1	37.5 31.3 31.3	0.297
Season (%) -Winter -Spring -Summer -Autumn	20 10 40 30	24 8 36 32	0 14.3 21.4 64.3	0.219
**1 year after birth visit (n)**	24	14	9	57
BMI (kg/m^2^)	27.7 ± 6.4	25.9 ± 6.5	30.4 ± 7.4	0.293
Fasting glycemia (mg/dL)	86.8 ± 9	91.4 ± 8.6	90.1 ± 7.3	0.265
Fasting insulin (µUI/mL)	8.0 ± 7.4	8.1 ± 4.1	8.7 ± 4.6	0.955
HbA1c (%)	5.3 ± 0.2	5.4 ± 0.2	5.4 ± 0.4	0.280
25-OH-D (ng/mL)	22.9 ± 7.8 ^a^	24.9 ± 5.3 ^a^	32 ± 10.5 ^b^	**0.017**
25-OH-D categories (%) -Deficiency (<20 ng/mL) -Insufficiency (20–29.9 ng/mL) -Sufficiency (≥30 ng/mL)	37.5 50 12.5	7.7 69.2 23.1	11.1 33.3 55.6	**0.035**
HOMA-IR	1.7 ± 1.7	1.8 ± 0.9	2 ± 1.2	0.894
HOMA-B	203.8 ± 441.8	109.9 ± 58.9	113.8 ± 45.1	0.619
MD adherence score	7.8 ± 1.6	8.7 ± 2.1	7.5 ± 1.6	0.249
Daily walking/cycling (min.)	27.0 ± 20.6	30.6 ± 20.3	26.7 ± 25.9	0.871
Season (%) -Winter -Spring -Summer -Autumn	26.1 17.4 0 56.5	21.4 21.4 21.4 35.7	22.2 11.1 11.1 55.6	0.432

BMI: body mass index; 25OH-D: 25-hydroxyvitamin D; HOMA-IR: homeostasis model assessment of insulin resistance index; HOMA-B: homeostasis model assessment of β-cell function; MD: Mediterranean diet; min. (minutes). Different superscripted letters mean significant differences (*p* < 0.05) according to an ANOVA followed by Duncan’s post hoc test. Factors related to 25-OH-D levels at every time point.

**Table 2:** Multivariate linear regression analysis: association between 25-OH-D at every time point and different variables.

	**25-OH-D Levels at Baseline**		
	**R^2^ 0.258**		
Independent variables	B	95% CI	*p*
Age (years)	0.092	−0.150–0.333	0.455
Group -Control -Lifestyle -Insulin	Ref. 1.415 1.679	- −1.671–4.502 −2.419–5.778	0.367 0.420
BMI before pregnancy (kg/m^2^)	−0.197	−0.404–0.010	0.061
HOMA-IR	−0.029	−0.825–0.766	0.942
MD adherence score	0.533	−0.083–1.149	0.090
Vitamin D supplements use	3.131	0.086–6.175	**0.044**
Season -Winter -Spring -Summer -Autumn	Ref. 0.360 10.948 2.371	−2.761–3.481 7.368–14.528 −0.922–5.665	0.692 **<0.001** 0.157
	**25-OH-D levels at antenatal visit**		
	R^2^ 0.419		
Age (years)	−0.299	−0.641–0.043	0.085
Group -Control -Lifestyle -Insulin	Ref. 6.787 6.256	2.540–11.034 1.040–11.472	**0.002** **0.019**
Weight gain in pregnancy (kg)	0.237	−0.098–0.527	0.164
HOMA-IR	−0.046	−0.383–0.292	0.789
Season -Winter -Spring -Summer -Autumn	Ref. −1.951 13.869 6.688	−6.657–2.755 9.246–18.492 1.541–11.834	0.413 **<0.001** **0.011**
	**25-OH-D levels at postpartum visit**		
	R^2^ 0.140		
Age (years)	0.090	−0.195–0.374	0.532
Group -Control -Lifestyle -Insulin	Ref. 1.341 5.538	−1.747–4.428 1.556–9.521	0.390 **0.007**
BMI (kg/m^2^)	−0.097	−0.377–0.183	0.494
HOMA-IR	−0.819	−2.002–0.365	0.172
Season -Winter -Spring -Summer -Autumn	Ref. 2.527 1.669 2.455	−3.073–8.127 −2.242–5.579 −1.631–6.541	0.372 0.398 0.235
	**25-OH-D levels at 1 year after birth**		
	R^2^ 0.298		
Age (years)	0.085	−0.480–0.651	0.761
Group -Control -Lifestyle -Insulin	Ref. 1.136 8.831	−4.763–7.035 2.467–15.195	0.699 **0.008**
BMI (kg/m^2^)	−0.210	−0.740–0.320	0.427
HOMA-IR	−0.187	−2.717–2.343	0.882
Season -Winter -Spring -Summer -Autumn	Ref. −1.692 5.288 −0.187	−9.239–5.855 −5.516–16.093 −3.034–10.044	0.652 0.328 0.284

BMI: body mass index; 25OH-D: 25-hydroxyvitamin D; HOMA-IR: homeostasis model assessment of insulin resistance index; MD: Mediterranean diet.

## Data Availability

The data is contained within the article.
